# Identifying and Targeting Mortality Disparities: A Framework for Sub-Saharan Africa Using Adult Mortality Data from South Africa

**DOI:** 10.1371/journal.pone.0071437

**Published:** 2013-08-14

**Authors:** Benn Sartorius, Kurt Sartorius

**Affiliations:** 1 School of Public Health, Faculty of Health Sciences, University of the Witwatersrand, Johannesburg, South Africa; 2 Faculty of Commerce, Law and Management, University of the Witwatersrand, Johannesburg, South Africa; Rollins School of Public Health, Emory University, United States of America

## Abstract

**Background:**

Health inequities in developing countries are difficult to eradicate because of limited resources. The neglect of adult mortality in Sub-Saharan Africa (SSA) is a particular concern. Advances in data availability, software and analytic methods have created opportunities to address this challenge and tailor interventions to small areas. This study demonstrates how a generic framework can be applied to guide policy interventions to reduce adult mortality in high risk areas. The framework, therefore, incorporates the spatial clustering of adult mortality, estimates the impact of a range of determinants and quantifies the impact of their removal to ensure optimal returns on scarce resources.

**Methods:**

Data from a national cross-sectional survey in 2007 were used to illustrate the use of the generic framework for SSA and elsewhere. Adult mortality proportions were analyzed at four administrative levels and spatial analyses were used to identify areas with significant excess mortality. An ecological approach was then used to assess the relationship between mortality “hotspots” and various determinants. Population attributable fractions were calculated to quantify the reduction in mortality as a result of targeted removal of high-impact determinants.

**Results:**

Overall adult mortality rate was 145 per 10,000. Spatial disaggregation identified a highly non-random pattern and 67 significant high risk local municipalities were identified. The most prominent determinants of adult mortality included HIV antenatal sero-prevalence, low SES and lack of formal marital union status. The removal of the most attributable factors, based on local area prevalence, suggest that overall adult mortality could be potentially reduced by ∼90 deaths per 10,000.

**Conclusions:**

The innovative use of secondary data and advanced epidemiological techniques can be combined in a generic framework to identify and map mortality to the lowest administration level. The identification of high risk mortality determinants allows health authorities to tailor interventions at local level. This approach can be replicated elsewhere.

## Introduction

The achievement of the Millenium Development Goals (MDG) in many developing countries has been compromised because of the persistent nature of many health problems combined with limited resources [1]–[2]. Excess adult mortality in Sub Saharan Africa (SSA) has been neglected due to a focus on improving infant and child survival [3]–[4], as well as a lack of data and a failure to exploit available databases for monitoring and evaluating purposes [5]–[7]. Estimates of adult mortality in the region vary depending on which data source was used, the assumptions made and the methodology employed. However, it is evident that adult mortality in some countries of the region are still the highest in the world [8]–[9]. SSA, moreover, is the epicentre of the HIV/AIDS pandemic [10]–[11], maternal mortality remains a problem [12] and the impact of non-communicable disease is expected to increase [11], [13]–[14]. The high level of adult mortality and morbidity in SSA has also impacted negatively on the economy and healthcare resources of the region. In particular, HIV/AIDS has reduced the availability and productivity of working aged adults [14]–[18]. Given the disturbing adult mortality data in the region, as well as its impact on the economies of its countries, the lack of adult mortality research and policy development is surprising.

A standard framework for the identification and analysis of geographical areas of high risk mortality and population attributability of prominent determinants, is an important policy issue [19]. This importance is underlined by the limited healthcare resources of developing countries which often precludes the implementation of population-wide intervention programs in favour of targeted high risk areas or “hotspots” (local areal units with significantly higher than expected mortality) at a regional or local level [20]. Recent advances in data availability, software and analytic methods, have created new opportunities to address this challenge. These advances, moreover, have improved the identification, analysis and mapping of health inequality from national to local level [21]–[24].

This paper develops a generic framework for SSA to identify and assess adult mortality and its associated determinants. Using South African national survey data as an example, the generic framework first identifies the spatial risk of high adult mortality clusters at four levels. The framework then quantifies the relationship between adult mortality and a range of high impact determinants before developing a mortality reduction index to show the effect of the removal of the most attributable factors. This is especially important given the limited resources for healthcare interventions in the region [25]. Finally, the discussion supports the wider application of the proposed generic framework in SSA rather than expanding on the social determinants of adult mortality.

## Materials and Methods

### Study Area

**Figure 1:**
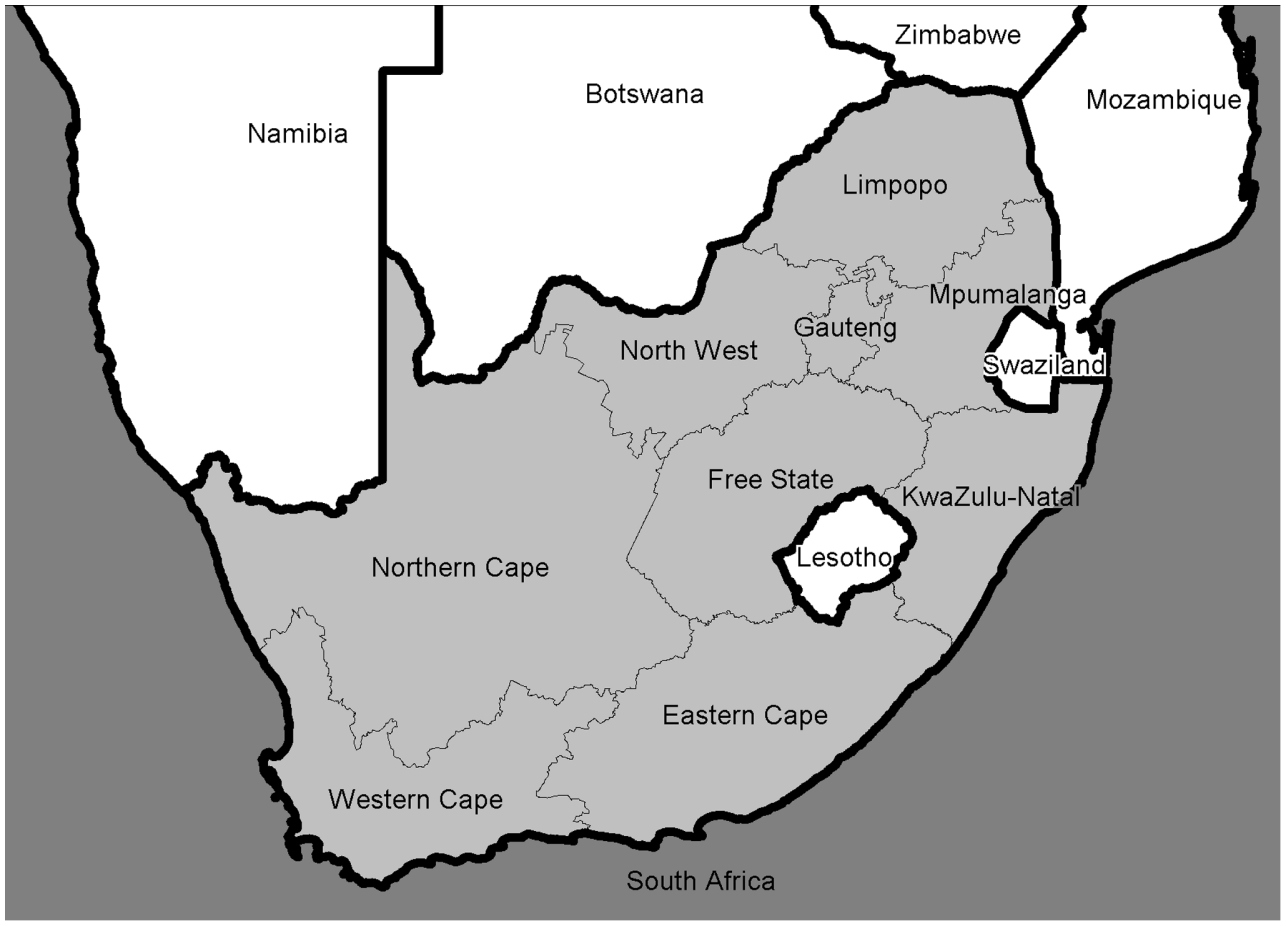
Map of South Africa, with provinces and neighbouring countries.

South Africa is administratively divided into nine secondary regions (provinces) responsible for health service delivery (Figure 1) that are further sub-divided into 52 tertiary regions. At the time of the study these consisted of 46 district municipalities and 6 metropolitan districts (currently South Africa’s provinces are sub-divided into 44 district municipalities and 8 metropolitan districts). These districts are then further disaggregated into 248 quaternary areas (local municipalities which are the lowest administrative unit).

### Data Sources

The data were drawn from the random cross sectional national survey run by Statistics South Africa in 2007. These data included information regarding demographic indicators (such as mortality) and socio-economic data including education, employment, household assets, as well as access to facilities and services [26]. The survey sample covered 274 348 dwelling units across all the provinces and attained a response rate of 93.9% [27]. The South African Statistics Council [28] also found the reported demographic data to be plausible when compared to other censuses.

This secondary data analysis focused on adults aged 15 to 59 years. Mortality was based on interviewee reported mortality of household members in the preceding 12 months. Individual level mortality data were not provided due to confidentiality reasons. Thus, aggregated adult mortality at the smallest available aggregation (quaternary level) was used. Demographic (age, gender and population group), social (marital status, disability, education, household goods) economic activity (employment, income, occupation) and household service (water, sanitation, refuse removal, settlement type) data were extracted. Household level demographic indicators were calculated based on individual characteristics. Additional data, regarding district level antenatal HIV sero-prevalence in 2007, were extracted from the District Health Barometer for 2007/2008 [29]. Mortality data from the vital registration system (available at the provincial level) were also used for validation purposes and cause-of-death fractions.

### Statistical Analyses

#### Identification of significant spatial ‘hotspots’ using difference and equivalence testing

Adult mortality proportions were calculated for each district and local municipality (sub-district) by dividing the observed number of deaths by the total population in a given areal unit. To identify areas in which the mortality proportion was significantly above average, we first constructed the exact 95% confidence intervals (CI) for each proportion using the binomial distribution of the observed number of deaths [30]. Areal unit mortality was considered significantly above average if the lower 95% CI limit (α = 0.025) of the mortality proportion for that district or local municipality was above the overall national average [31]. Standardised mortality ratios (SMRs) were also estimated by dividing observed deaths by the expected number of deaths if the national average mortality incidence applied to that areal unit’s total adult population.

#### Potential determinants of identified spatial hotspots

This component of the analysis compared significantly high risk local municipalities (unit of analysis) with the remaining local municipalities with regards to significant differences in the breakdown of various individual, household and community or local municipality level determinants. Given the aggregated nature of the mortality data, an ecological modelling approach was employed.

Various multilevel determinants assessed included: age, gender, education, population group, marital status, employment status and income, household services, and antenatal HIV sero-prevalence. The outcome was defined at the individual level and whether they resided in one of the identified local municipality mortality hotspots. The ecological relationship between adult mortality hotspots (dichotomous) and various determinants was assessed using preliminary bivariate associations, clustering on the unit of analysis (quaternary level) to correctly adjust the standard errors and not overestimate significance. Covariates significant at the 10% level were then incorporated into a multivariable model. The multivariable model is formulated as follows with Y_ij_ being a dichotomous outcome classification (1 = local municipality hotspot, 0 = non-hotspot) for individual _i_ in local municipality _j_:
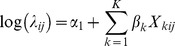
where *x*_1*ij*_,…,*x_kij_* represents the various covariates, *α*_1_ is the constant (or intercept) and *β_k_* are the regression coefficients. We employed a Poisson modelling approach (with log link) for binary (dichotomous) outcome data [32] to estimate risk ratios (RR) directly as odds ratios (OR) could overestimate coefficient “risk” given a prevalent outcome. Use of ordinary Poisson regression with binary data may however result in standard errors that are too conservative (i.e. overestimate significance) due to under-dispersion from fitting a Poisson regression model to binomial data [32]–[33]. However, one can use a Poisson approach with robust standard errors to give correct standard errors (deals with variance overestimation) [34]. We therefore also used robust error variance in our model and again clustered on the unit of analysis (quaternary level) to correctly adjust the standard errors and not overestimate significance.

#### Population Attributable Fractions (PAF)

We also assessed the degree to which small area unit exposure (prevalence) to a particular variable (e.g. access to water and sanitation) impacted on mortality. This could provide an indication for policy makers about what intervention(s) to prioritise and the potential impact of removing such an exposure. We linked determinant coefficient estimates with the actual prevalence of exposure to those determinants within the lowest level areal units. The following standard formula for calculating an attributable fraction for each determinant was based on its prevalence of exposure (p_e_) in a given areal unit, as well as the exponentiated model coefficient (risk ratio[RR]) for that determinant:
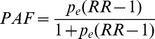



#### Software

Analysis was carried out in STATA 12.0 SE and WinBUGS. Maps were constructed in MapInfo Professional.

## Results

### Primary Level (National)

The study sample comprised 640 761 adults aged 15–59 with a mean age of 32.6 years (std.dev. 12.4). The overall adult mortality proportion was approximately 145.2 deaths per 10 000 population (95% CI: 142.3, 148.2), with males having a significantly (p-value<0.001) higher mortality proportion of 155.1 (95% CI: 150.8, 159.6) compared to females at 135.9 (95% CI: 132.0, 140.0) per 10 000 population respectively.

### Secondary Level (Province)

**Figure 2:**
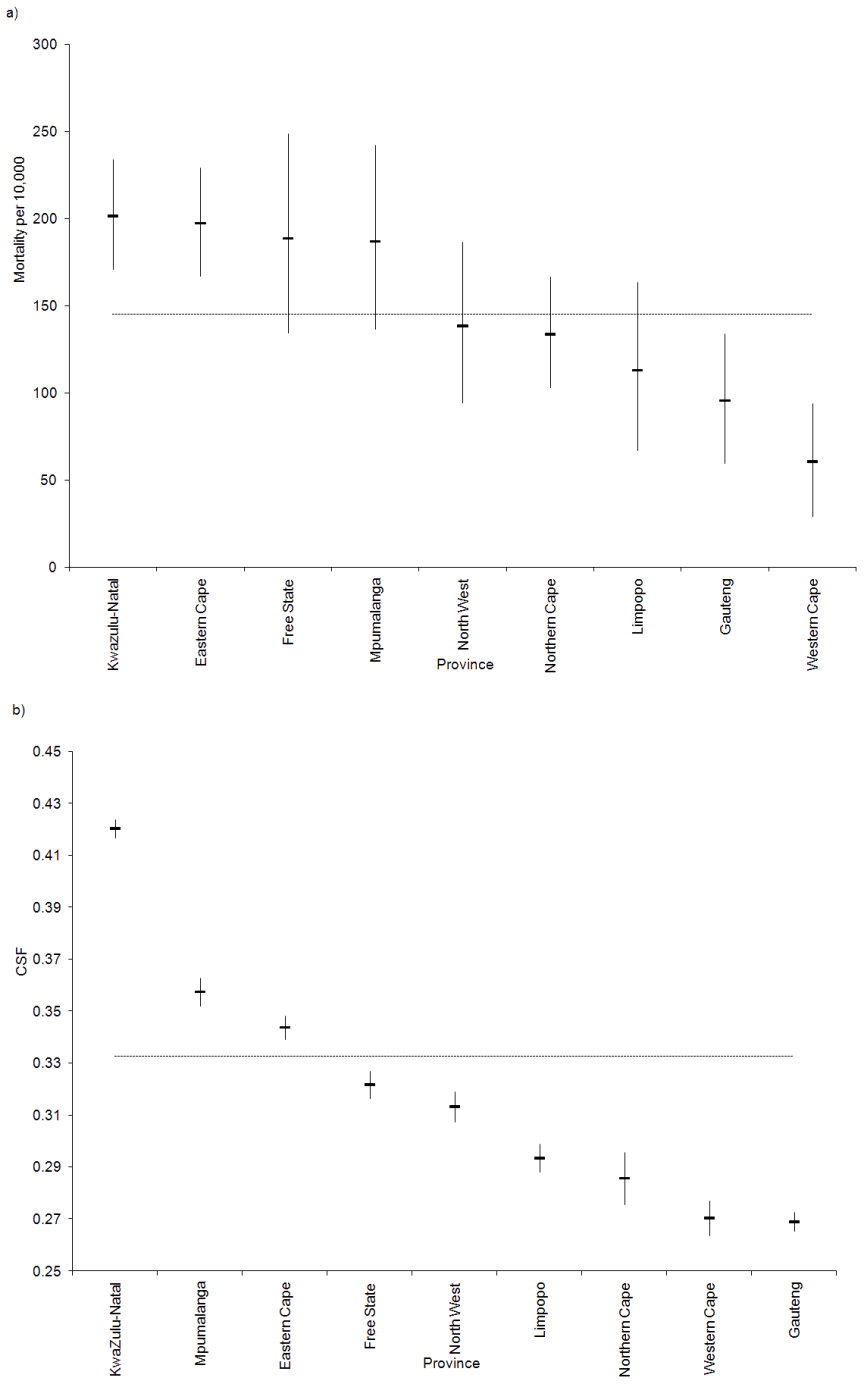
a) Descending all-cause adult mortality proportions (survey) and b) cause-specific fractions attributed to infectious causes (vital registration) at the secondary level (province), South Africa, 2007. [CSF = cause specific fraction].

Secondary level adult mortality differs significantly across South Africa’s nine provinces (Figure 2a). Two neighbouring provinces, namely, Kwazulu-Natal and the Eastern Cape have significantly higher adult mortality compared to the national average. Conversely, the Western Cape and Gauteng displayed much lower levels. The top five broad causes of deaths among adults aged 15to59 years (based on vital registration estimates available at provincial or national level only) by province are presented in Table 1. The leading cause of death among adults in all provinces was attributed to infectious causes (largely HIV/TB). The remaining four of the top five cause of death by province were generally (with minor variations in ranking) attributed to external, diseases of the respiratory system, unknown (R00-R99) and diseases of the circulatory system. Deaths attributed due to infectious causes strongly and significantly correlate (Pearson correlation coefficient [ρ] = 0.841, p-value<0.01) with provincial level estimates of all-cause adult mortality based on survey estimates (Figure 2a,b). Western Cape was the only province where neoplasm (cancer) related mortality appeared in the top five ranking (∼12% of adult deaths and ranked 3^rd^).

**Table 1:** Top five broad causes of death by province, South Africa, 2007.

Province	Cause	Cause specificdeath count	Totaldeaths	CSF
Eastern Cape	Certain infectious and parasitic diseases (A00–B99)	14505	42190	34.38%
	External (V01–Y98)	5536	42190	13.12%
	Diseases of the respiratory system (J00–J99)	5060	42190	11.99%
	Ill defined (R00–R99)	4995	42190	11.84%
	Diseases of the circulatory system (I00–I99)	3257	42190	7.72%
Free State	Certain infectious and parasitic diseases (A00–B99)	9982	31031	32.17%
	Diseases of the respiratory system (J00–J99)	6001	31031	19.34%
	Ill defined (R00–R99)	3307	31031	10.66%
	External (V01–Y98)	2538	31031	8.18%
	Diseases of the circulatory system (I00–I99)	2511	31031	8.09%
Gauteng	Certain infectious and parasitic diseases (A00–B99)	15149	56330	26.89%
	External (V01–Y98)	8852	56330	15.71%
	Diseases of the respiratory system (J00–J99)	7722	56330	13.71%
	Ill defined (R00–R99)	7569	56330	13.44%
	Diseases of the circulatory system (I00–I99)	5112	56330	9.08%
KwaZulu-Natal	Certain infectious and parasitic diseases (A00–B99)	32921	78323	42.03%
	Ill defined (R00–R99)	8709	78323	11.12%
	External (V01–Y98)	8697	78323	11.10%
	Diseases of the respiratory system (J00–J99)	8625	78323	11.01%
	Diseases of the circulatory system (I00–I99)	5519	78323	7.05%
Limpopo	Certain infectious and parasitic diseases (A00–B99)	7910	26947	29.35%
	Diseases of the respiratory system (J00–J99)	4542	26947	16.86%
	Ill defined (R00–R99)	4151	26947	15.40%
	External causes of morbidity and mortality (V01–Y98)	2613	26947	9.70%
	Diseases of the circulatory system (I00–I99)	1980	26947	7.35%
Mpumalanga	Certain infectious and parasitic diseases (A00–B99)	10695	29918	35.75%
	Diseases of the respiratory system (J00–J99)	5198	29918	17.37%
	External causes of morbidity and mortality (V01–Y98)	2877	29918	9.62%
	Diseases of the circulatory system (I00–I99)	2332	29918	7.79%
	Diseases of the blood and immunity disorders (D50–D89)	2103	29918	7.03%
North West	Certain infectious and parasitic diseases (A00–B99)	7835	25009	31.33%
	Diseases of the respiratory system (J00–J99)	4257	25009	17.02%
	Ill defined (R00–R99)	2873	25009	11.49%
	External causes of morbidity and mortality (V01–Y98)	2741	25009	10.96%
	Diseases of the circulatory system (I00–I99)	2540	25009	10.16%
Northern Cape	Certain infectious and parasitic diseases (A00–B99)	2217	7760	28.57%
	Diseases of the respiratory system (J00–J99)	1126	7760	14.51%
	External causes of morbidity and mortality (V01–Y98)	994	7760	12.81%
	Ill defined (R00–R99)	980	7760	12.63%
	Diseases of the circulatory system (I00–I99)	683	7760	8.80%
Western Cape	Certain infectious and parasitic diseases (A00–B99)	4722	17466	27.04%
	External causes of morbidity and mortality (V01–Y98)	4012	17466	22.97%
	Neoplasm’s (C00–D48)	2128	17466	12.18%
	Diseases of the circulatorysystem (I00–I99)	1977	17466	11.32%
	Diseases of the respiratorysystem (J00–J99)	1190	17466	6.81%

### Tertiary Level (District)

**Figure 3:**
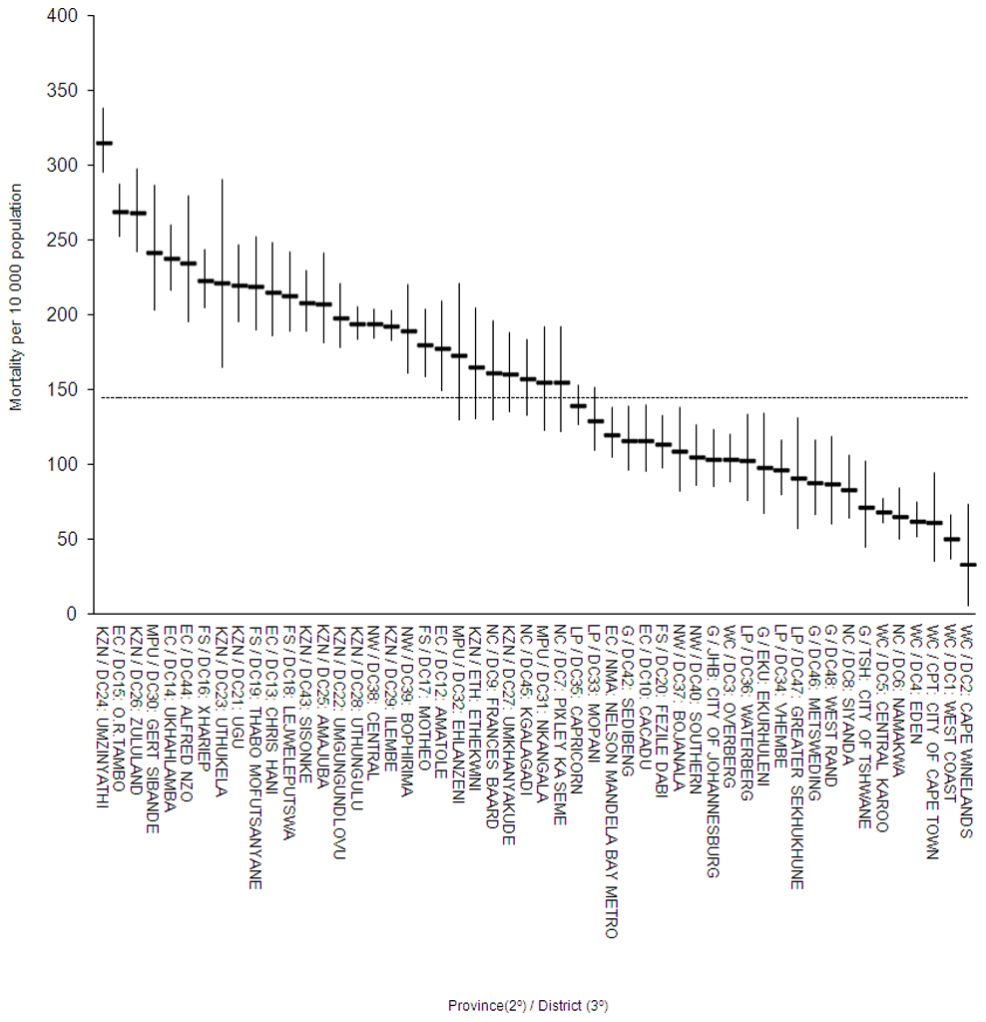
Descending district level adult mortality proportions with 95% confidence intervals and highlighting significantly high or low tertiary (districts) areas, South Africa, 2007 (KZN Kwazulu-Natal: EC: Eastern Cape; MPU: Mpumalanga; FS: Free State; NW: North West; NC: Northern Cape; LP: Limpopo; G: Gauteng; WC: Western Cape). Dashed line represents the national average.

Tertiary level adult mortality differs significantly different across South Africa’s 52 districts (Figure 3) in line with the levels reflected in the nine provinces. Particularly, 16 districts in Kwazulu-Natal and the Eastern Cape (mostly) show the highest levels while 12 districts in the Western Cape and Gauteng generally have the lowest levels.

### Quaternary Level (Local Municipality)

**Figure 4:**
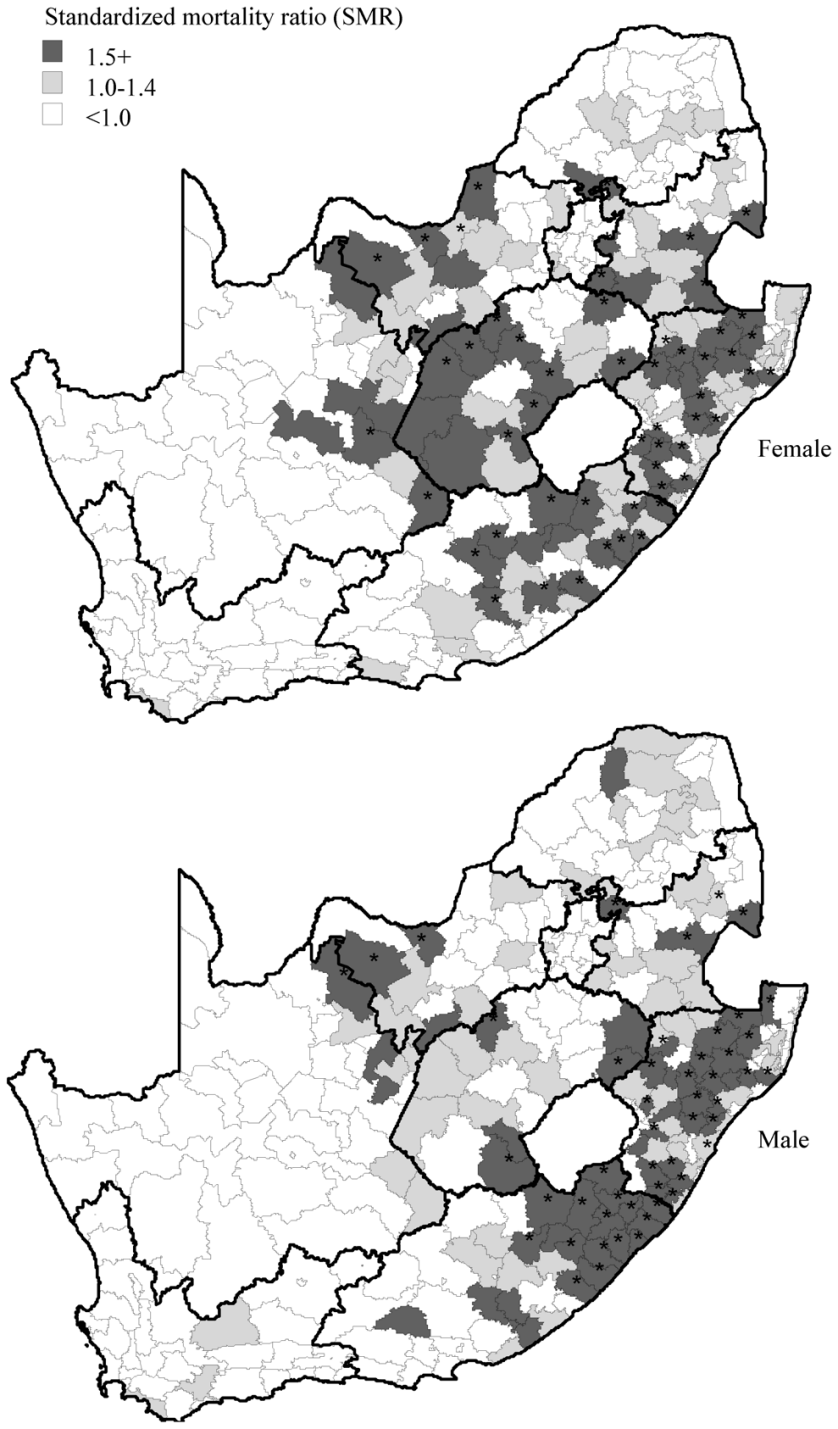
Adult mortality risk by gender at the quaternary (local municipality) level, South Africa, 2007. Districts with significant excess risk (p<0.05) are highlighted (*).

A total of 67 mortality “hotspots” were identified in South Africa’s 248 local municipalities. Further analysis by gender (Figure 4) shows the biggest clusters of adult mortality for both males and females in Kwazulu-Natal and the Eastern Cape. Conversely, the Western Cape and Gauteng recorded none. A highly significant (p-value<0.01) level of correlation (ρ = 0.65) was observed between female and male adult mortality. A strong cluster of female mortality was observed in seven local municipalities in the Free State that also displayed a significantly higher risk of pregnancy related mortality based on vital registration data (259 per 100,000) than all the other provinces except for the Eastern Cape.

### Common Determinants and their Impact on Mortality in Quaternary Level “Hotspots” based on an Ecological Approach

A comparison of the distribution of potential determinants, for high risk versus remaining local municipalities, indicated significant differences for a range of individual and household characteristics (Table 2). High risk local municipalities exhibited significant individual level differences including a lower age, a higher proportion of females and no income and less stable marital patterns. Differences in education were only marginally significant (p-value = 0.128). At household level, high risk local municipalities showed a higher proportion of households with no annual income, a higher mean number of household occupants, lower asset status, lower levels of formal housing and less access to basic household services. Death rates during pregnancy were also markedly elevated in the high risk local municipalities. As antenatal HIV sero-prevalence data were not available at local municipality level, data at tertiary level were used to show that a strong degree of correlation was displayed between adult mortality rates and antenatal HIV sero-prevalence (ρ = 0.48, p-value<0.01).

**Table 2:** Bivariate multilevel determinants for high risk adult mortality areas using an ecological framework, South Africa, 2007.

	High risk local municipalities		Remaining local municipalities		
Factors	N (168 774)	(95% CI)	N (471 983)	(95% CI)	*P*-value i
**Individual characteristics**					
Mean age	168 774	26.80 (26.72,26.87)	471 983	29.00 (28.95,29.04)	<0.001
Percentage female	168 774	53.14 (52.97,53.32)	471 983	51.29 (51.17,51.40)	<0.001
Percentage African	168 774	89.60 (89.49,89.70)	471 983	72.00 (71.90,72.10)	0.010
Education	155 010		422 296		0.128
Percentage with no education		11.22 (11.09,11.35)		9.20 (9.12,9.27)	
Percentage with primary level		40.57 (40.38,40.77)		34.23 (34.11,34.35)	
Percentage with secondary level		46.69 (46.49,46.89)		53.72 (53.60,53.85)	
Percentage with tertiary level		1.52 (1.47,1.57)		2.85 (2.81,2.89)	
Marital status	155 010		422 296		<0.001
Percentage union (non-poly)		22.15 (22.00,22.31)		29.29 (29.18,29.40)	
Percentage union (poly)		0.055 (0.046,0.064)		0.046 (0.041,0.051)	
Percentage never married		71.43 (71.26,71.60)		64.73 (64.62,64.85)	
Percentage widower/widow		5.23(5.14,5.31)		4.02 (3.98,4.07)	
Percentage with serious disability	155 010	2.97 (2.90,3.03)	422 296	2.27 (2.24,2.31)	0.006
Percentage with no monthly income	168 774	45.86 (45.68,46.03)	471 983	41.23 (41.12,41.35)	<0.001
**Household characteristics**					
Mean occupants	168 774	3.50 (3.48,3.52)	471 983	2.88 (2.87,2.89)	<0.001
Percentage living in a modern dwelling	168 774	41.47 (41.14,41.80)	471 983	50.58 (50.39,50.77)	0.002
Mean absolute asset count	153 722	2.94 (2.93,2.96)	418 764	3.58 (3.57,3.59)	0.017
Services	168 774		471 983		
Percentage with no water service provider		25.59 (25.29,25.88)		10.15 (10.03,10.26)	0.002
Percentage with no toilet facilities		12.84 (12.61.13.06)		5.23 (5.15,5.32)	0.003
Percentage with no refuse removal		8.63 (8.45.8.84)		4.69 (4.60,4.77)	0.013
Percentage with none of the above		2.62 (2.5.2.73)		0.66 (0.63,0.69)	0.001
Percentage with no annual income	168 774	6.97 (6.80,7.15)	471 983	5.45 (5.36,5.54)	0.018
**Local municipality deaths in pregnancy per** **100 000 live births**	168 774	240.54 (173.68,307.41)	471 983	54.35 (35.52,73.19)	<0.001
**District antenatal HIV sero-prevalence (%)**	168 774	31.67 (30.10,33.24)	471 983	24.46 (23.01,25.90)	<0.001

i Robust logistic regression for categorical variables and linear regression for comparison of means were employed. Note these formulations were used in place of standard χ2 and t- tests respectively to allow for clustering on the unit of analysis (quaternary level) and thus correctly adjust the standard errors (robust) and not erroneously overestimate significance.

Further analysis illustrates the multivariable adjusted association of selected identified determinants (Table 3). Not being in a formal marital union, low socio-economic status (SES), lack of basic household services and high district antenatal HIV sero-prevalence (> = 30%) were significantly more frequent among individuals in the high risk areal units based on the bivariate analysis. Not being in a formal union, lack of basic household services and antenatal HIV sero-prevalence remained significant following multivariable adjustment. HIV was the most attributable factor followed by not being in a formal union and lastly low SES.

**Table 3:** Example of a targetable framework of modifiable determinants for high risk adult mortality areas using a multivariable Poisson ecological approach and including population attributable fractions, South Africa, 2007.

Factors	Unadjusted RR(95% CI)	Adjusted RR(95% CI)	Prevalence ofexposure	PAF^i^(95% CI)
Individual: Male gender	1.13 (1.07,1.19)	1.13 (1.07,1.19)	0.52	0.06 (0.04,0.09)
Individual: Not in a formal union^ii^	1.28 (1.16,1.41)	1.40 (1.26,1.56)	0.62	0.20 (0.14,0.26)
Individual/Household: Low socio-economic statusiii	1.43 (0.97,2.12)	1.70 (1.15,2.51)	0.39	0.21 (0.06,0.37)
Household: No basic household service^iv^	2.05 (1.27,3.29)	3.19 (1.59,6.39)	0.01	0.03 (0.01,0.07)
Local municipality: metropolitan area	0.56 (0.06,5.07)	–	–	–
District: Antenatal HIV sero-prevalence > = 30 percent	2.63 (1.28,5.42)	3.98 (1.38,11.45)	0.42	0.56 (0.14,0.81)

i population attributable fraction.

ii never married, separated, divorced or widowed.

iii based on individual level education status, monthly income and household assets (e.g. fridge, radio etc).

iv no water service, sanitation and refuse disposal.

### The Removal of Attributable Factors

**Figure 5:**
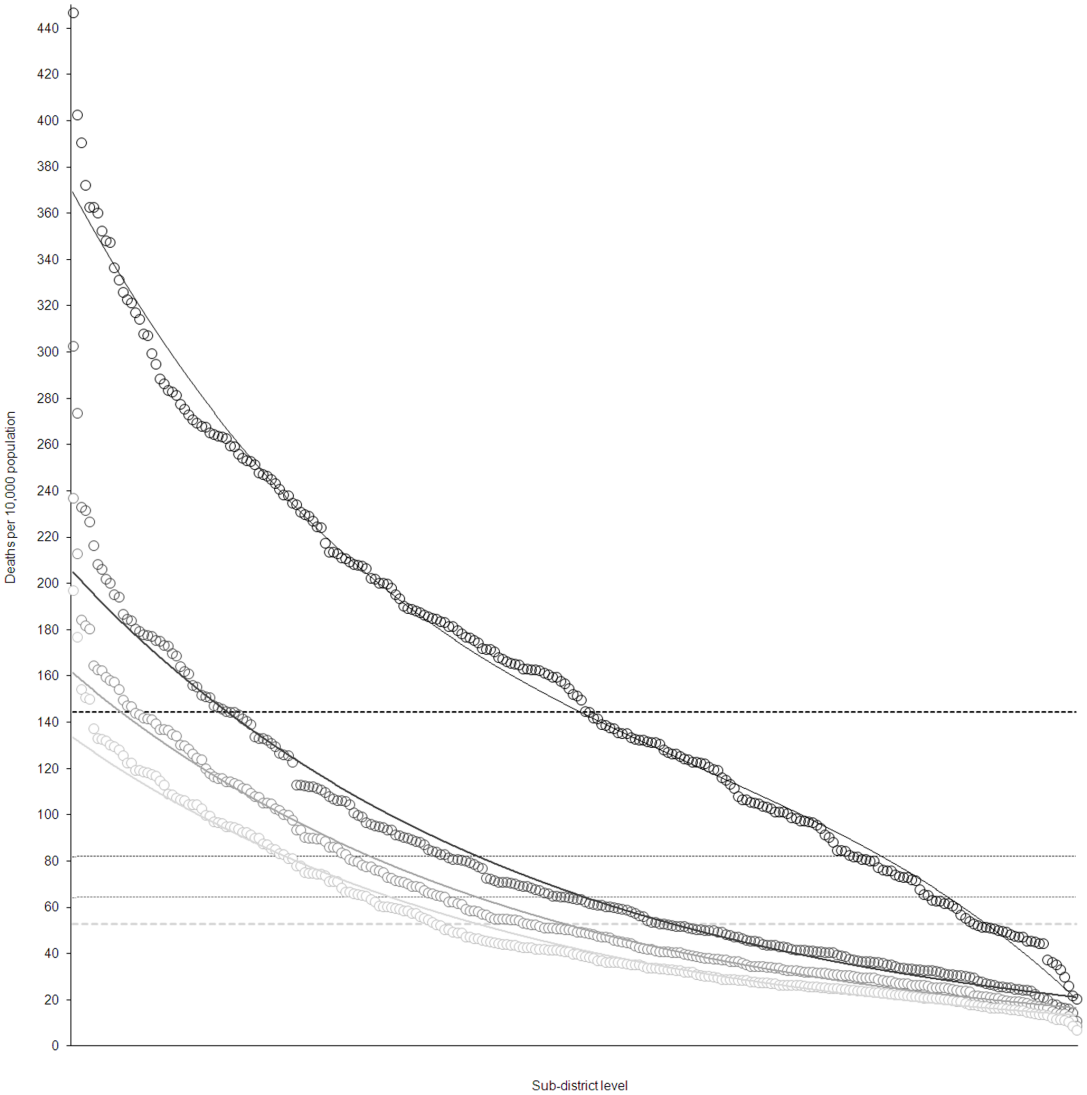
Mortality proportion by quaternary unit: observed and projections based on attributable factor removal based on quaternary unit level prevalence of exposure, South Africa, 2007. Local municipalities are ordered from highest to lowest mortality: black curve represents the observed mortality; dark grey curve represents adjusted mortality following removal of the most attributable local municipality factors, grey curve following removal of the secondary most attributable factors, light grey line following removal of the tertiary most attributable factors. Overall average reductions are displayed using horizontal dash lines of the same colour.

Antenatal HIV sero-prevalence was the most attributable factor in the high risk quaternary (local municipality) areas with the highest PAF in 37 of the 67 (55%) high risk quaternary areas. For the remaining high risk areas, low SES appeared to be the other most attributable (28 local municipalities or 42%). Low SES ranked as the second most attributable factor in the high risk municipalities (34 local municipalities or 51%) followed by lack of formal union (30 municipalities or 45%). Lack of formal union status and male gender appeared to be the third most attributable ranked factors (34 and 25 local municipalities or 51 and 37% respectively). Results suggest that primary level attributable factors at local municipality level account for approximately 44% (95% CI: 42–48%) of adult deaths. Secondary attributable factors appeared to account for 21% (95% CI: 20–22%). The impact of removing these determinants, based on the prevalence of the primary and secondary most attributable factors in the individual quaternary units, is illustrated in Figure 5. If the most attributable in selected quaternary units are removed, a reduction in overall mortality from the observed 145 to 82 deaths per 10,000 is predicted. If we also remove the second and third most attributable risk factors, the overall mortality level potentially reduces to ∼53 deaths per 10,000 i.e. a projected net total reduction of ∼92 deaths per 10,000 population. Notably we observe a stabilisation of the lowest unit mortality disparity as high risk determinants are removed.

**Figure 6:**
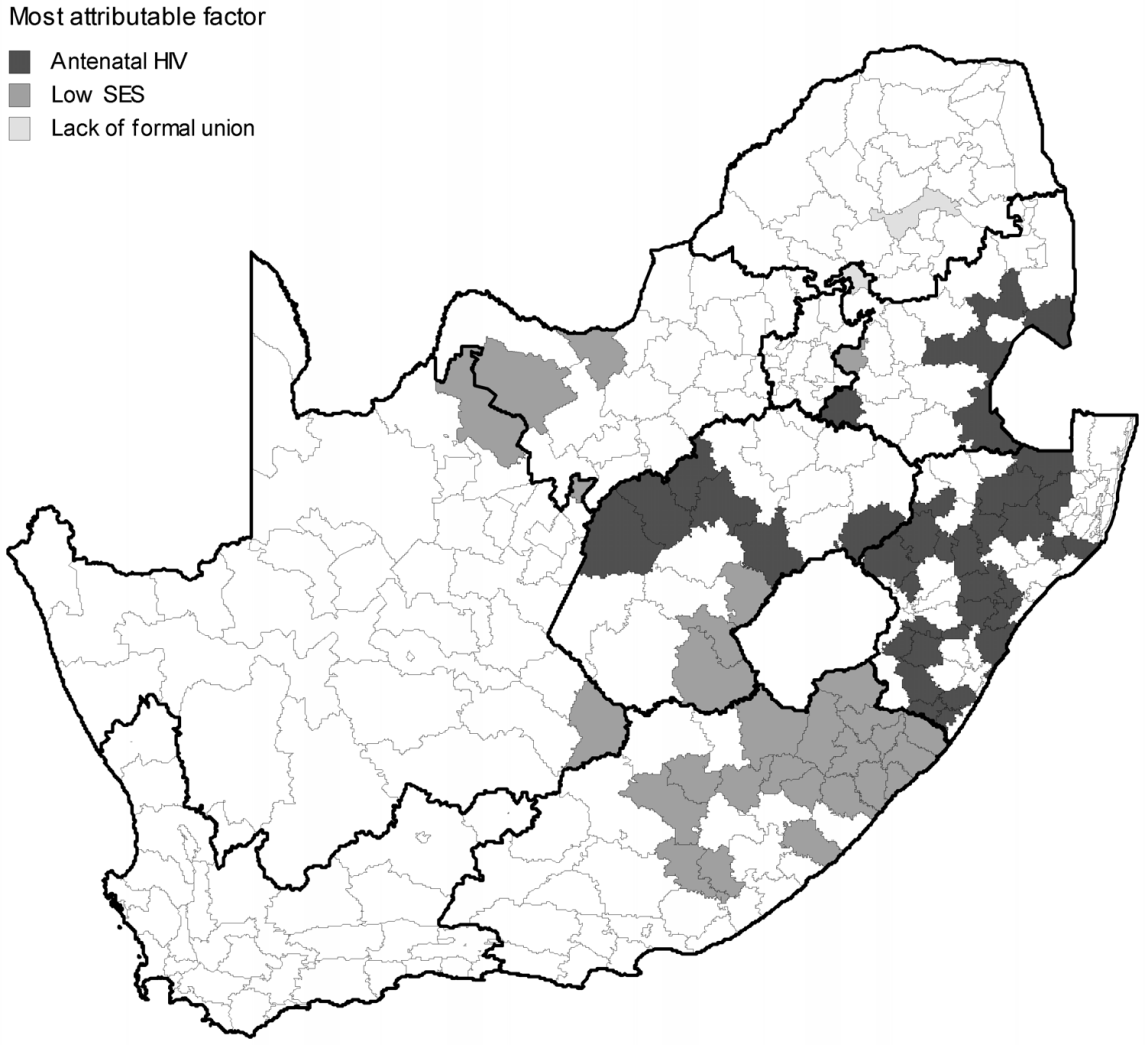
Distribution of primary attributable factors in significant high risk adult mortality local municipalities, South Africa, 2007. Provincial boundaries are shown in bold.

A “policy maker” map is developed showing the distribution of the most attributable risk factors in the high risk local municipalities (Figure 6). Findings suggest that HIV is the more prominent factor in Kwazulu-Natal and Free State while low SES appears to feature more strongly in the Eastern Cape. This could easily be extended to the secondary and tertiary most attributable factors.

## Discussion

### Usefulness of the Proposed Framework and Applicability in Other Settings

The integration of spatial disparities of mortality, and the estimation of modifiable determinants using secondary data to most effectively guide resources at sub-national levels, has received little attention despite its prominence as a policy issue [19]. This framework extends the use of prediction models to estimate mortality at a sub-national level in a developing country context [35]. The proposed framework is also easily adaptable to other settings and could also be used for mortality in other age groups, especially for example children <5 years.

The primary purpose of the study was to present a reusable generic framework that could be used in the SSA setting. To illustrate the framework we employed common determinants only to illustrate the properties of the proposed framework rather than explore the detailed causal relationships between adult mortality and related determinants. This study, therefore, does not present a thorough conceptual framework to assess adult mortality nor does it attempt to discuss the identified adult mortality determinants and related policy implications.

### Disaggregating Adult Mortality and Identification of Hotspots

The national (primary) adult mortality rate in South Africa is consistent with SSA, largely due to the general impact of the HIV/AIDS pandemic in the region [6] although South Africa has the highest HIV/AIDS infected population in the world with 5.5 million infected individuals [36]. The estimated adult mortality rate of 145 per 10,000, based on the survey, was slightly higher than those based on “comprehensive” vital registration estimates (120 per 10,000). The proportionally higher mortality rates of males is consistent within the SSA although the female mortality in the region appears to be increasing sharply [4]. This increasing level of maternal mortality is reflected in our data and has been associated with the HIV/AIDS pandemic, poverty, a lack of maternal education and poor health facilities in many rural areas [12]. A stronger concentration of significant higher risk local municipality areas for females, compared to males, is also illustrated in one province (Free State). National vital registration data also suggest that this province has the highest level of pregnancy related mortality.

The adult mortality results delivered by our generic framework are well supported by other studies in South Africa that show significant differences across its nine provinces [37]–[38]. Interestingly, a distribution of adult mortality at provincial level as a result of communicable disease only, shows an identical pattern of differences across the nine provinces illustrating the continuing influence of the HIV/AIDS pandemic on overall mortality levels. In particular, Kwazulu-Natal and the Eastern Cape, show markedly higher levels of adult mortality than the Western Cape and Gauteng [38].

Adult mortality in South Africa’s 52 districts (tertiary) shows a further level of disaggregation that is consistent with the distribution of adult mortality at regional (provincial level). Adult mortality in certain districts in Kwazulu-Natal and the Eastern Cape is in excess of 300 deaths per 10,000 whilst it is less than 50 in some districts of the Western Cape. Finally, we disaggregated adult mortality to the lowest administrative level (quaternary) mortality to identify 67 high risk local municipality (quaternary) areas. These results are consistent with other mortality studies that reflect high levels of adult mortality at local municipality level in Kwazulu-Natal, the Eastern Cape, the Free State and Mpumalanga [38].

### Modifiable Determinants of Mortality and Impact of Removal

In order to demonstrate the use of the generic framework, we used a range of well discussed determinants to illustrate attributable factors influencing adult mortality in the region. Population attributable fractions (PAF) for specific predictors are useful to help guide policymakers in planning public health interventions [39]–[40]. Our model, therefore, combines a spatial analysis and high impact determinants to examine the association of individual, household and local municipality (“community”) characteristics on adult mortality hotspots by using an ecological type design. A recent study using demographic and health surveys (DHS) data from 20 countries in sub-Saharan Africa has shown that local context independently affects the mortality of residents, in addition to individual and household level determinants [41]. Our study confirms the enduring importance of contextual higher level factors (e.g. HIV, low SES) as important determinants of adult health and mortality in the region. The continuing impact of the HIV/AIDS epidemic on adult mortality has been documented elsewhere, for example [42]. In further support of the generic framework, other potentially high impact determinants can be evaluated for policy development purposes. A generic mortality framework for the SSA region appears feasible and can be readily adapted to other age-groups and related outcomes.

### The Removal of Attributable Factors at a Population Level

The proposed framework demonstrates the impact of some common determinants of mortality in South Africa including a lack of basic services, low education, age, income, gender, marital status and HIV sero-prevalence [43]. The results, therefore, show that the proposed framework can effectively target “hotspots” at the lowest areal level, as well as identify possible association based on exposure prevalence within these units. A PAF estimates the proportion of a given outcome (in this case mortality) in a given population that would “theoretically” not have occurred if none of the individuals had been exposed to a given risk factor. The proposed intervention is thus not at the traditional individual (or “downstream”) level but rather at a societal or population level. Whether the full theoretical population level gain can be achieved in practice is difficult and would require large scale support and policy change at a governmental level to effect population level reductions [44]–[45]. Furthermore distal determinants of health (for example national, political, and socio-cultural factors) indirectly influence health by acting on the more proximal factors [46]. Certain factors used in this study could potentially be more distal in nature rather than directly modifiable proximal causal factors. Health policies aimed at preventable factors (i.e. modifiable by public health) should play a key role in the overall assessment of health systems [46]. Utilizing more directly “modifiable” proximal risk factors in the proposed framework would also be a suggested approach in future policy related applications. The possible impact of confounding on coefficient estimates and related overestimation of the associated PAF cannot also be discounted and is discussed further in the limitations section below. The acuteness of geographical location, as well as the range of mortality determinants, illustrate the need to integrate healthcare interventions with other programs like rural development and education [17]. By targeting the top two most attributable determinants in this empirical example (local HIV, low SES), the framework demonstrates the potential to “theoretically” reduce adult mortality levels from 145 to 53 deaths per 10,000 population which is more in line with levels in more developed settings [4]. This approach has a definite application in other settings and for other outcomes in SSA and can assist policy makers to more effectively guide resources at sub-national levels to reduce overall national mortality in line with MDGs. By selective targeting of the most attributable determinants in high risk areas (e.g. Figure 6), there is the potential to reduce overall national mortality levels to those in line with more developed settings.

### Data Exploitation in SSA

The use of spatial mortality risk maps at a local level is especially important in SSA due to a limitation in national health care budgets, as well as donor funds [36]. The innovative use of databases in the region, like the DHS combined with national and survey data, offer much potential to improve our knowledge of adult mortality in the region [6]. The use of spatial analysis can also be used to map high risk mortality at local level in order to target interventions in a country with limited resources [47]. Death registration data, however, needs to be improved in the SSA to pinpoint differential interventions for cause-specific outcomes, as well as show changing trends of lifestyle and chronic disease types due to economic growth and population ageing in the region [48].

Certain limitations and potential systematic errors were identified by Statistics SA and the South African Statistical Council when reviewing the survey data [27]. This secondary data analysis is limited by the data that were included in the primary community survey. The consistency of our findings with other national estimates would suggest that our findings are fairly reliable and valid. Given the ecological (aggregated) nature of the data, caution should be taken with interpretation of the direct causal inferences found in our bivariate and multivariable analysis. The data were extracted down to the smallest administrative areal unit available (namely local municipality) which should reduce the ecological effect. For PAF estimation if one or more factors act as confounders between exposure and health outcome, this may lead to biased crude PAF estimates and there is a need for adjustment when estimating the PAF [49]. Multivariable models allow taking into account confounding factors [50] and the model needs to be as complete as possible. However, even when using adjusted relative risk estimates, PAF estimates can be biased if there are unaccounted confounding factors leadings to overestimation of PAF [51].
